# The association of cellulitis incidence and meteorological factors in Taiwan

**DOI:** 10.1017/S0950268819000323

**Published:** 2019-03-11

**Authors:** Ren-Jun Hsu, Chia-Cheng Chou, Jui-Ming Liu, See-Tong Pang, Chien-Yu Lin, Heng-Chang Chuang, Cheng-Keng Chuang, Hsiao-Wei Wang, Ying-Hsu Chang, Po-Hung Lin

**Affiliations:** 1 Graduate Institute of Life Sciences, National Defense Medical Center, Taipei, Taiwan; 2 Cancer Medicine Center of Buddhist Hualien Tzu Chi Hospital, Tzu Chi University, Hualien, Taiwan; 3 Department of Pathology and Graduate Institute of Pathology and Parasitology, Tri-Service General Hospital, National Defense Medical Center, Taipei, Taiwan; 4 Division of General Surgery, Department of Surgery, Taoyuan General Hospital, Ministry of Health and Welfare, Taoyuan, Taiwan; 5 Department of Medicine, National Yang-Ming University, Taipei, Taiwan; 6 Division of Urology, Department of Surgery, Taoyuan General Hospital, Ministry of Health and Welfare, Taoyuan, Taiwan; 7 Division of Urology, Department of Surgery, Chang Gung Memorial Hospital at Linkou, Taoyuan, Taiwan; 8 Department of Pediatrics, Hsinchu MacKay Memorial Hospital, Hsinchu, Taiwan; 9 Division of Infection Diseases, Department of Internal Medicine, Shuang Ho Hospital, Taipei Medical University, New Taipei City, Taiwan; 10 Graduate Institute of Clinical Medical Sciences, College of Medicine, Chang Gung University, Taoyuan, Taiwan

**Keywords:** Cellulitis, epidemiology, meteorological factors, NHIRD

## Abstract

Cellulitis is a common infection of the skin and soft tissue. Susceptibility to cellulitis is related to microorganism virulence, the host immunity status and environmental factors. This retrospective study from 2001 to 2013 investigated relationships between the monthly incidence rate of cellulitis and meteorological factors using data from the Taiwanese Health Insurance Dataset and the Taiwanese Central Weather Bureau. Meteorological data included temperature, hours of sunshine, relative humidity, total rainfall and total number of rainy days. In otal, 195 841 patients were diagnosed with cellulitis and the incidence rate was strongly correlated with temperature (*γ*_S_ = 0.84, *P* < 0.001), total sunshine hours (*γ*_S_ = 0.65, *P* < 0.001) and total rainfall (*γ*_S_ = 0.53, *P* < 0.001). The incidence rate of cellulitis increased by 3.47/100 000 cases for every 1° elevation in environmental temperature. Our results may assist clinicians in educating the public of the increased risk of cellulitis during warm seasons and possible predisposing environmental factors for infection.

Cellulitis is a common infectious disease of the skin and soft tissues, which is a frequent presentation in outpatient and emergency departments. The incidence of cellulitis in the USA was reported in 2006 to be approximately 2500 cases per 100 000 patient years and occurred most frequent in middle-aged males and older adults [1]. A more recent systematic review suggests that overall worldwide mortality rates attributed to ‘cellulitis and abscess’ range from 0.7% to 1.8% [2]. Several predisposing factors have been noted for cellulitis particularly chronic renal and liver disease which may reduce host immunity, as well as conditions such as varicose veins and lymphoedema are associated with higher risk of infection [3]. The most common organisms isolated from cellulitis lesions in individuals with normal immunity are the group A Streptococci and *Staphylococcus aureus*.

Seasonal and meteorological factors may also increase the risk of skin infection as shown by a recent study of lower limb cellulitis from Australia which reported a higher incidence in summer and autumn seasons [4]. Moreover, the odds of hospital admission for patients with cellulitis in the USA increase with higher environmental temperatures in a dose-response fashion [5]. In the present study, we used a 13-year dataset to analyse possible associations between the incidence rate of cellulitis and meteorological parameters in Taiwan.

Taiwan is located in the Western Pacific Ocean; the Tropic of Cancer crosses the southern part of the country and defines the climate type as tropical and subtropical maritime. The population of Taiwan is *c.* 23 million people in a total area of *c.* 36 000 km^2^; the north–south distance being roughly 400 km. Meteorological seasons are, spring: March–May; summer: June–August; autumn: September–November and winter: December–February. Monthly meteorological data obtained from the Taiwanese Central Weather Bureau from 27 weather stations distributed across the country and outlying islands were recorded for analysis. Data included temperature, total sunshine hours, relative humidity, total rainfall and total number of rainy days (Supplementary Table 1). We used the average 1-month meteorological data value from all weather stations to evaluate the relationship between weather and the incidence of cellulitis.

The study was approved by the Institutional Review Board of Tri-Service General Hospital, National Defense Medical Centre (IRB No. B-104-14). All registries and medical records in the dataset were anonymised and digitised to avoid the need for consent. We used the database of the Taiwanese public health insurance system [Longitudinal Health Insurance Database (LHID) 2000 of the National Health Insurance Research Dataset (NHIRD)] which covers >98% of the population with approximately 23 million individual registries. The dataset contained all medical claims data from hospitals along with patients’ demographic data. The LHID 2000 comprises the original data of 1 million individuals randomly sampled from the year 2000 by Taiwanese National Health Research Institutes. The NHIRD registry maintains the registration data of each beneficiary within the National Health Insurance Programme from 2000 to 2013. There was no significant difference in gender distribution (*χ*^2^ = 1.74, df = 1, *P* = 0.187) between the patients in the LHID 2000 and the original NHIRD.

Patients receiving either ambulatory or emergency care with a diagnosis of cellulitis according to the International Classification of Diseases, Ninth Revision, Clinical Modification (ICD-9-CM) codes 37601, 5283, 681, 6810, 68100, 6811, 68110, 6819, 682, 6820, 6821, 6822, 6823, 6824, 6825, 6826, 6827 and 6828 in LHID 2000 from 2001 to 2013 were included in the study (Supplementary Table 2). Patients with cellulitis prior to 2001 were excluded as were those residing in counties without a weather station. Once the first episode of cellulitis was recorded and entered into the monthly incidence data, the patient was removed from the dataset to avoid the confounder of recurrent infections. The monthly incidence rate was calculated based on the remaining population in the month and correlated with the corresponding monthly meteorological data.

Spearman's rank correlation was used to evaluate the relationship between the cellulitis incidence rate and each meteorological factor. Correlation coefficients (*γ*_S_) falling between 0.80 and 1.00 were defined as very strong. Coefficients representing strong, moderate, weak or very weak correlations correspond to the values of 0.60 to 0.79, 0.40 to 0.59, 0.20 to 0.39 and 0.00 to 0.19, respectively; these data were entered into a linear regression model to estimate linkage between each factor and incidence rate. All tests were two-sided with an *α* value of 0.05 to reduce type-I errors. All data preparation and analysis were performed with SAS 9.2 statistical software (version 9.4; SAS Institute Inc., Cary, NC, USA).

The study flowchart is illustrated in Figure 1. From 2001 to 2013, 266 620 patients were entered into the dataset. After exclusions, a total of 195 841 cellulitis patients were enrolled in the study comprising 51.7% males (mean age 41.3 ± 20.78) and 48.2% females (42.55 ± 20.53) (Supplementary Table 3).

**Fig. 1.**
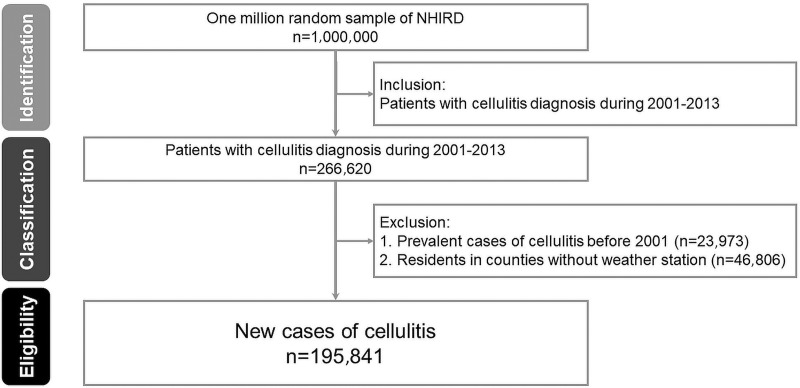
The flowchart of data extraction. A total of 195 841 patients with a first-time diagnosis of cellulitis from 2001 to 2013 were enrolled. NHIRD, National Health Insurance Research Dataset.

The average monthly meteorological factors with the corresponding monthly cellulitis incidence rates are shown in Table 1. The highest incidence rates were recorded in July, August and September, and the lowest in December, January and February. There was a very strong correlation of incidence rates with temperature (*γ*_S_ = 0.84, *P* < 0.001), a strong correlation with total sunshine hours (*γ*_S_ = 0.65, *P* < 0.001), a moderate correlation with total rainfall (*γ*_S_ = 0.53, *P* < 0.001) and a weak correlation with relative humidity and total number of rainy days. All meteorological data showed statistical significance.

**Table 1.** Average monthly incidence rate of cellulitis and meteorological factors in the 14-year study period

Month	Monthly incidence rate			Temperature (°C)			Total sunshine hours	Relative humidity (%)	Total rainfall (mm)	Total rain days
	Total	Male	Female	Mean	Max.	Min.				
January	122.47^a^	122.74^a^	122.18^a^	16.90^a^	21.9	10.6	128.48^a^	75.95	62.23^a^	8.29
February	112.68^a^	112.51^a^	112.87^a^	18.17^a^	23.5	10.8	125.81^a^	77.64	66.78^a^	7.78^a^
March	130.3	130.08	130.54	19.79	24.3	12.8	139.23	75.42	77.97	9.5
April	131.64	135.22	127.86^a^	22.94	26.8	17.1	128.83^a^	77.24	108.26	10.84
May	146.34	146.97	145.67	25.86	28.7	20.5	160.26	77.85^b^	213.51	12.09^b^
June	150.89	152.22	149.48	27.52^b^	29.8	21.6	167.2	79.31^b^	315.79^b^	13.80^b^
July	164.05^b^	167.31^b^	160.62^b^	28.72^b^	30.8	22.5	223.84^b^	77.14	291.61^b^	11.28
August	162.43^b^	164.47^b^	160.28^b^	28.48^b^	30.1	22.3	196.28^b^	78.31^b^	347.25^b^	13.52^b^
September	152.07^b^	155.07^b^	148.91^b^	27.39	30	21.2	171.5	77.44	283.08	11.52
October	146.14	147.75	144.44	24.92	28	19	175.30^b^	73.97^a^	101.41	6.51^a^
November	133.23	133	133.48	22.06	25.6	16.9	139	75.03^a^	95.65	7.87
December	127.19^a^	125.92^a^	128.53	18.41^a^	22.9	11.6	137.63	73.73^a^	71.05^a^	7.39^a^

a Lowest three values.

b Highest three values.

The incidence rate showed a significant trend correlating with temperature, the total number of sunshine hours and total rainfall (Supplementary Fig. 1). It was also noted that the cellulitis rate decreased year on year as a consequence of exclusion of repeat cases, and other factors, from the dataset. These trends were confirmed by the linear regression model which showed that average temperature (*β* = 3.47, *P* < 0.001), total sunshine hours (*β* = 0.30, *P* < 0.001), relative humidity (*β* = 1.16, *P* < 0.05), total rainfall (*β* = 0.06, *P* < 0.01) and total number of rainy days (*β* = 1.43, *P* < 0.001) were significantly associated with cellulitis incidence. The average monthly temperature proved to be the most significant factor showing an increase in cellulitis incidence of 3.47/100 000 according to each 1° rise in temperature.

Meteorological factors in Taiwan exhibit seasonal changes with high temperatures in summer and longer days of intense sunshine. There is increased rainfall due to typhoons primarily in late summer and autumn. In the winter, the sunshine days are shorter and the Northeast monsoon brings cold air which precipitates on mountains as persistent drizzle. Furthermore, the East-Asian rainy season (also known as plum rain) occurs in late spring and early summer. The highest rainfall and number of rainy days are significantly higher in late spring and summer giving a marginally higher relative humidity in the latter. Thus, the cellulitis incidence was directly correlated with season, being highest in summer and lowest in winter.

Several examples of the association of infectious diseases with seasonality have been described, citing a number of possible factors. These include the effect of different seasons on the survival of pathogens outside of the host due to environmental conditions such as temperature, humidity, exposure to sunlight, pH and salinity [6]. Studies have investigated the association of seasonality in subtropical and temperate climates with skin infections at different body sites, namely upper and lower extremities [3, 4, 7], fingers and toes [5] and general skin infection [8]. These studies concluded that the overall peak incidence of cellulitis occurred in warm weather, which is corroborated by the data presented here. Although we did not include data on the identity of the microbes isolated from our cellulitis cases, similar conclusions to those studies which addressed pathogen profiles and meteorological factors in subtropical areas may reasonably be drawn.

Meteorological conditions clearly have a profound effect on host behaviour, particularly in their choice of clothing and leisure activities, and have an indirect relationship between the incidence of cellulitis and weather. During high temperatures in summer in Taiwan people tend to wear short-sleeve clothing and short pants. Likewise, outdoor activities and swimming are popular, which may increase the probability of scrapes and grazes on uncovered areas which predispose individuals to skin and soft tissue infection. Summertime also corresponds to the new school semester in Taiwan where overcrowding of children in classrooms may facilitate the transmission of pathogens [9].

The age and sex-specific incidence rate of cellulitis was marginally higher in the 20–30 years old group compared with the less than 20 years old group, which may support a hypothetical link between infection rates and potentially outdoor exposure. Furthermore, analysis of the monthly cellulitis rate according to ICD-9-CM codes showed a higher incidence of infection in summer months at all body sites except for the head and neck. This suggests that factors other than climate may play an additional role in the pathophysiology of cellulitis of head and neck.

Finally, it has long been recognised that meteorological conditions, particularly temperature and humidity, may interfere with the skin barrier and promote bacterial growth [10]. In high-temperature sunny environments, people sweat more and the skin microclimate becomes more humid; rainfall in Taiwan is often sudden and heavy and these factors can facilitate the multiplication and spread of pathogens.

There are some limitations to this research. First, the study was based on a public health insurance registry dataset, and the pathogen data of individual cellulitis patients were not available, hence precluding analysis of associations of individual species with meteorological factors. Further analysis of the pathogen profiles from skin lesions is therefore warranted for future studies. Second, we used only the ICD-9 code to include cellulitis cases from ambulatory care or the emergency department and data on severity and complications of infections were not available. For minor skin infections, patients may prefer to seek topical treatments from the pharmacy, and such data were not included in the NHIRD. Likewise, patients with cellulitis on admission to hospital were excluded from the study and finally, specific host factors and comorbidities were not evaluated.

Nevertheless, this large-scale study correlating 13 years of meteorological data with the cellulitis incidence rate confirms its high seasonality in Taiwan, as observed elsewhere by others. This would be associated with increased hospitalisation and attendant medical costs [11]. Clinicians, and the general public, should therefore be more aware of the increased risk of cellulitis in the summer season in order to optimise treatment and take preventive action to decrease such risks.

## Supplementary material

For supplementary material accompanying this paper visit https://doi.org/10.1017/S0950268819000323. click here to view supplementary material
