# Supplementary material for: The association of cellulitis incidence and meteorological factors in Taiwan
Source: Epidemiol Infect. 2019 Mar 11;147:e138. doi: 10.1017/S0950268819000323 (PMC6518496; doi:10.1017/S0950268819000323)
Supplement: Supplementary file 1 [file S0950268819000323sup001.zip › Supplementary_Figure_1.docx]

**Supplementary Figure 1**

(A)


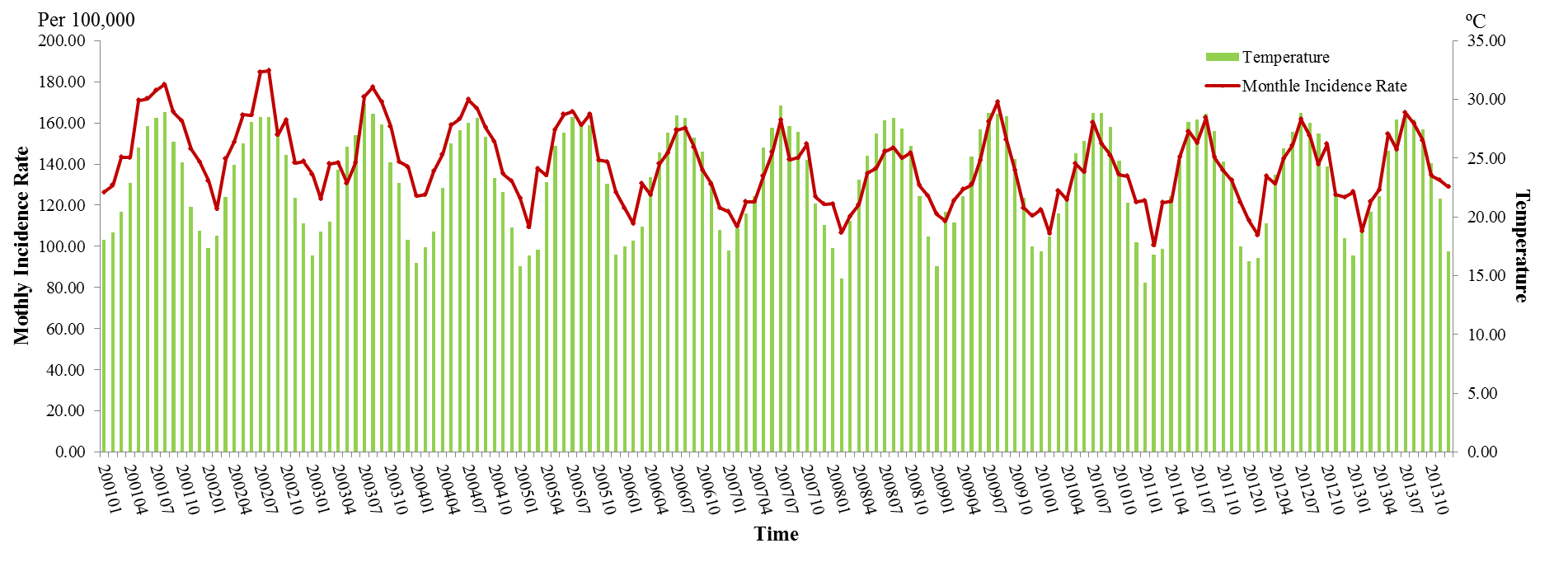


(B)


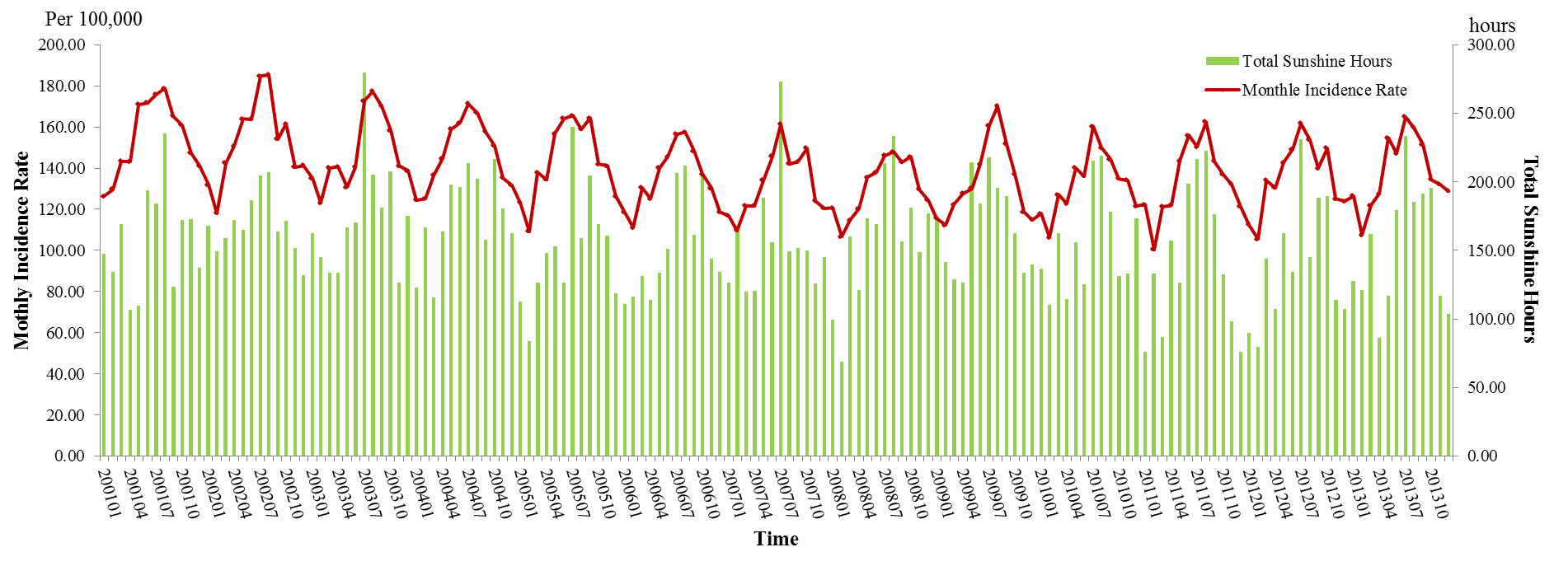


(C)


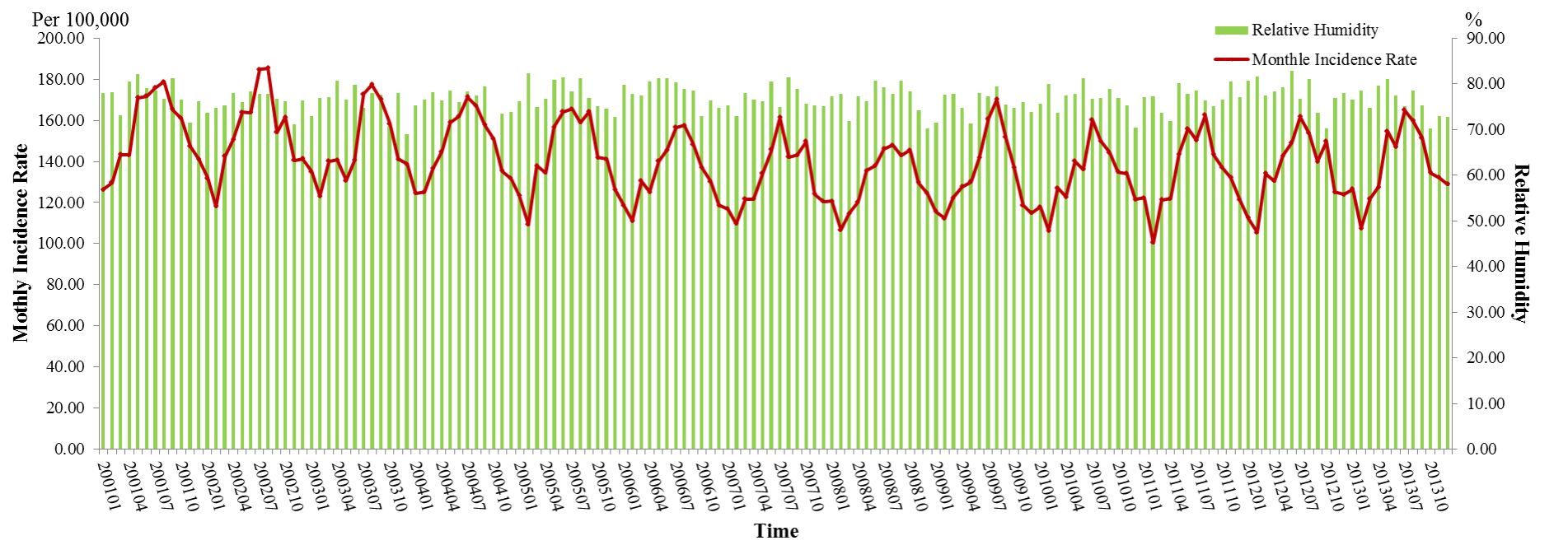


(D)


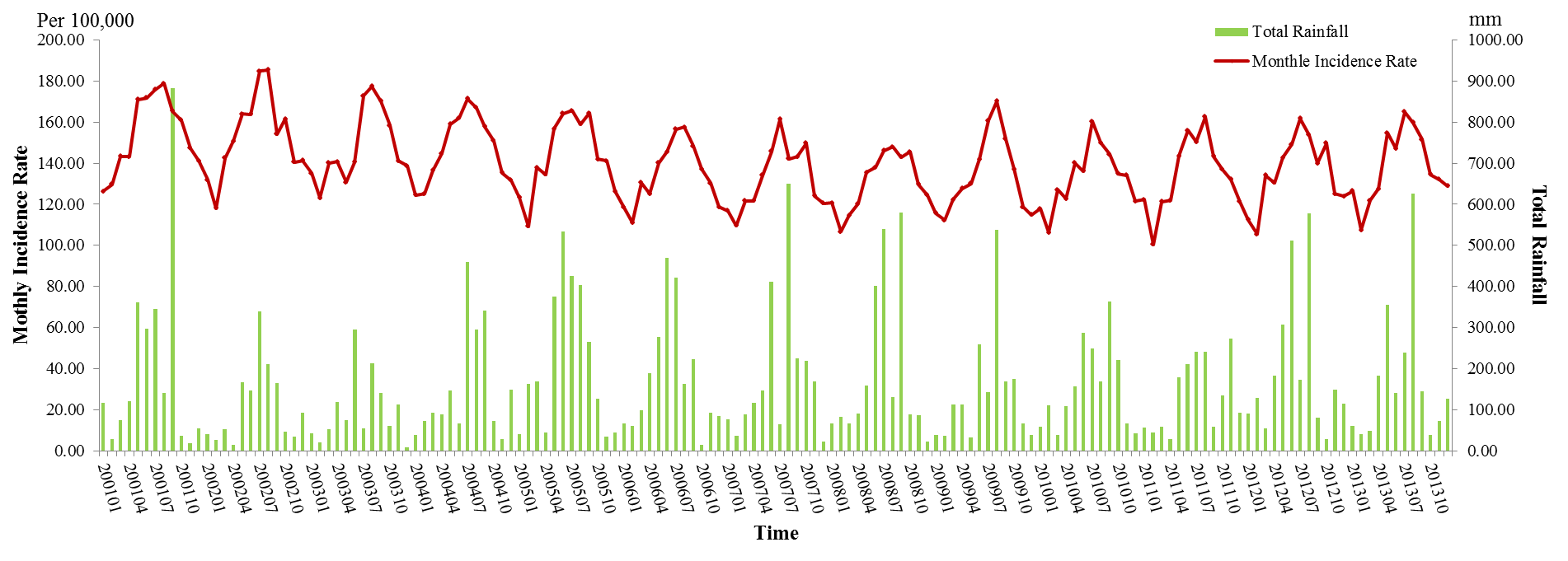


(E)


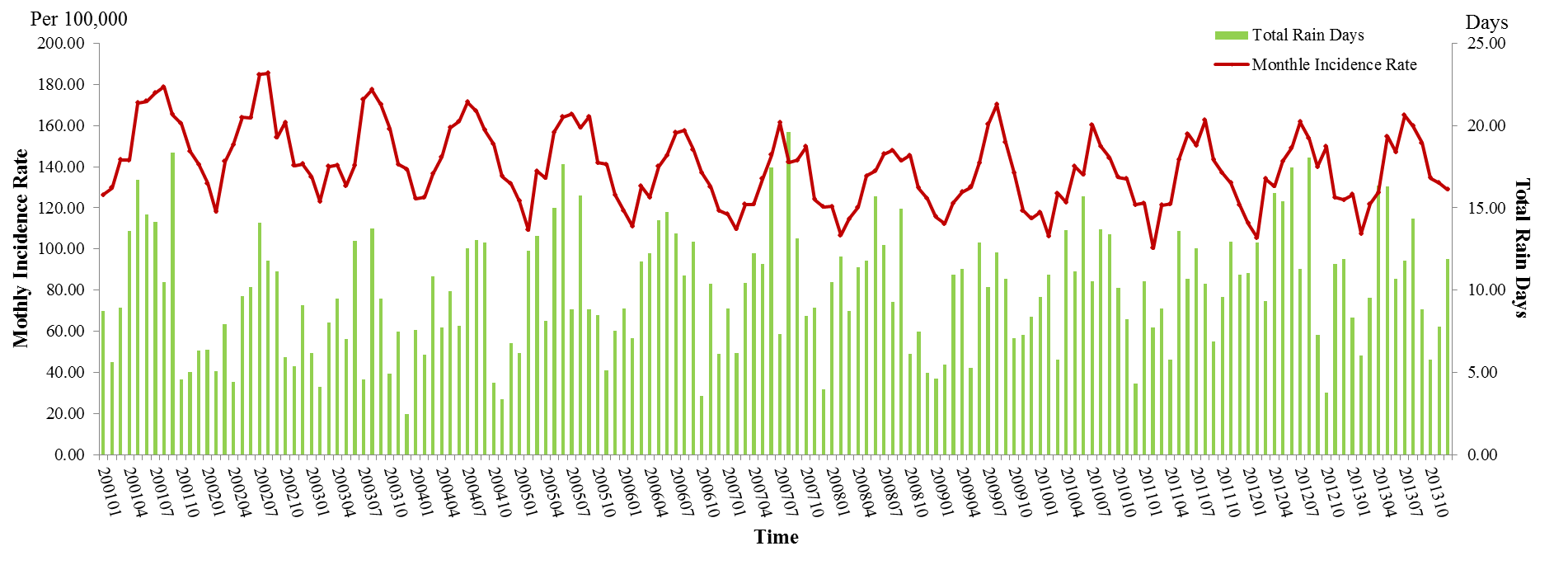


**Monthly meteorological data (bar chart) and corresponding incidence rate of cellulitis (line graph) during the study period 2001-2013. X-axis: time point. The marks are showed every three months. Left Y-axis: the incidence rate of cellulitis in 100000 population. Right Y-axis: the unit of meteorological data. (A) temperature (B) total sunshine hours (C) relative humidity (D) total rainfall (E) total rain days.**
